# Research on CTSA-DeepLabV3+ Urban Green Space Classification Model Based on GF-2 Images

**DOI:** 10.3390/s25133862

**Published:** 2025-06-21

**Authors:** Ruotong Li, Jian Zhao, Yanguo Fan

**Affiliations:** School of Oceanography and Spatial Information, China University of Petroleum, Qingdao 266580, China; s23160011@s.upc.edu.cn (R.L.); ygfan@upc.edu.cn (Y.F.)

**Keywords:** urban green space classification, semantic segmentation, DeepLabV3+, GF-2

## Abstract

As an important part of urban ecosystems, urban green spaces play a key role in ecological environmental protection and urban spatial structure optimization. However, due to the complex morphology and high degree of fragmentation of urban green spaces, it is still challenging to effectively distinguish urban green space types from high spatial resolution images. To solve the problem, a Contextual Transformer and Squeeze Aggregated Excitation Enhanced DeepLabV3+ (CTSA-DeepLabV3+) model was proposed for urban green space classification based on Gaofen-2 (GF-2) satellite images. A Contextual Transformer (CoT) module was added to the decoder part of the model to enhance the global context modeling capability, and the SENetv2 attention mechanism was employed to improve its key feature capture ability. The experimental results showed that the overall classification accuracy of the CTSA-DeepLabV3+ model is 96.21%, and the average intersection ratio, precision, recall, and F1-score reach 89.22%, 92.56%, 90.12%, and 91.23%, respectively, which is better than DeepLabV3+, Fully Convolutional Networks (FCNs), U-Net (UNet), the Pyramid Scene Parseing Network (PSPNet), UperNet-Swin Transformer, and other mainstream models. The model exhibits higher accuracy and provides efficient references for the intelligent interpretation of urban green space with high-resolution remote sensing images.

## 1. Introduction

With the acceleration of urbanization, urban green spaces have been continuously encroached upon, placing increasing pressure on urban ecosystems. Urban green spaces play a critical role in mitigating the urban heat island effect, purifying the air, and improving the microclimate [1,2,3,4,5,6]. They also serve as a fundamental component for achieving sustainable urban development. Against the backdrop of promoting “green development” and building “livable cities”, the ability to efficiently and accurately obtain urban green space classification data has become a prerequisite for the refined management of ecological space and the systematic optimization of green infrastructure [7,8].

The rapid development of remote sensing technology has provided effective tools for urban green space classification. Early studies primarily relied on satellite imagery from platforms such as Landsat and Sentinel [9,10,11,12], which offer strong temporal and spatial coverage. However, their limited spatial resolution hinders the detection of fragmented green areas and transitional boundaries within urban environments, making them insufficient for fine-scale classification tasks. In contrast, high spatial resolution remote sensing imagery offers a solid foundation for detailed urban green space classification. Among these, GF-2 (Gaofen-2) satellite imagery, with its sub-meter spatial resolution, has become an important data source for high-precision green space mapping [13,14,15]. Nevertheless, this also imposes higher demands on classification methodologies. Traditional remote sensing classification methods primarily include pixel-based and object-based approaches. Pixel-based methods are easy to implement and computationally efficient, and have been widely applied in early urban green space extraction tasks. However, when dealing with high-resolution imagery, these methods are prone to salt-and-pepper noise and discontinuities along object boundaries, which compromise classification consistency [16]. While object-based methods can alleviate pixel-level noise to some extent in fine urban vegetation classification, they still face challenges in high-resolution remote sensing applications, such as difficulty in selecting appropriate segmentation algorithms, complex feature extraction, and potential loss of fine spatial details [17,18]. Therefore, there is an urgent need for efficient and intelligent automated methods for urban green space classification to enable rapid perception and dynamic monitoring of urban green space patterns.

In recent years, deep learning methods have been widely applied in the field of remote sensing image segmentation due to their superior feature extraction capabilities [19,20,21,22]. Currently, deep learning-based semantic segmentation models for remote sensing imagery can be broadly categorized into two groups. The first group consists of models based on traditional convolutional neural networks (CNNs), such as the Fully Convolutional Network (FCN) [23], U-Net [24], and the DeepLab series [25,26]. These models extract hierarchical semantic information through successive convolutional operations and have demonstrated excellent pixel-level classification accuracy and strong algorithmic robustness in various remote sensing tasks, including water body detection, object extraction, and land surface classification [27,28,29]. Numerous enhancements have been proposed to further improve the performance of these CNN-based architectures. For instance, Shi et al. [30] incorporated the CBAM attention module into the U-Net framework and developed a water body segmentation approach applicable to multi-source remote sensing data. Men et al. [19] introduced CRAUNet by combining residual connections with a channel attention mechanism, which effectively improved segmentation accuracy in complex urban green space environments. Zhang and Zhao [31] proposed an improved DeepLabV3+ architecture by optimizing the atrous spatial pyramid pooling (ASPP) module and introducing an attention-based feature fusion module (AFFM), resulting in better segmentation performance under complex background conditions. Among these approaches, DeepLabV3+ has demonstrated particularly strong performance in semantic segmentation tasks involving natural scenes. Its multi-scale feature extraction mechanism significantly enhances the ability to capture objects across varying spatial scales, while the encoder–decoder structure facilitates more effective reconstruction of spatial features [32,33,34]. Nevertheless, most existing studies have concentrated on optimizing the encoder component, with limited emphasis on enhancing the capacity of the decoder to reconstruct high-dimensional features and recover fine spatial details [35,36]. This architectural imbalance constrains the potential for further advancements in fine-grained remote sensing classification.

The second category consists of semantic segmentation models based on the Transformer architecture, which fundamentally rely on the self-attention mechanism to capture global contextual relationships. The Vision Transformer (ViT) [37] was the first to introduce the Transformer architecture into computer vision tasks and has demonstrated strong performance in image segmentation. For instance, Qin et al. [38] proposed a ViT-based model for tunnel defect segmentation, enabling the unified detection of various defects such as cracks and water stains. Wang et al. [39] developed a remote sensing classification approach that integrates ViT with self-supervised pretraining, resulting in significantly improved classification accuracy and validating the effectiveness of ViT in representing multi-scale features in remote sensing imagery. However, in high spatial resolution remote sensing applications, such models often suffer from high parameter complexity and elevated computational costs [40]. To mitigate these limitations, a series of improved Transformer-based models have been proposed, including SegFormer [41] and Swin Transformer [42]. These models incorporate hierarchical feature pyramids and shifted window attention mechanisms, thereby enhancing multi-scale feature representation in remote sensing imagery and gaining popularity in semantic segmentation tasks. For example, Wang et al. [43] introduced an enhanced SegFormer model combining a multi-scale feature fusion network (MSF-FFN) with CoordAttention to extract winter wheat planting regions. Wu and Zhang [22] developed a hybrid framework, Swin-CFNet, which integrates Swin Transformer with convolutional layers for fine-scale urban green space classification, achieving an overall accuracy of 98.3% on high spatial resolution imagery. Despite notable improvements in segmentation performance, such models still require substantial computational resources, which limits their applicability in resource-constrained environments.

In summary, CNN-based models are limited by their constrained receptive fields, while Transformer-based approaches typically involve high computational complexity. To address the practical needs of high spatial resolution urban green space classification, a tailored GF-2-based dataset was constructed in this study, and an improved model—CTSA-DeepLabV3+—was proposed by incorporating a Contextual Transformer [44] module and the SENetv2 attention mechanism [45]. The main contributions of this study are summarized as follows:

1. A high spatial resolution urban green space dataset was constructed based on GF-2 satellite imagery and manually annotated, providing a reliable basis for training and evaluation.
2. A Contextual Transformer module was introduced into the decoder of DeepLabV3+ to enhance the capacity for capturing complex spatial structures and delineating fine-scale object boundaries. Meanwhile, SENetv2 was integrated to strengthen channel-level feature discrimination and suppress irrelevant responses, thereby improving classification precision.
3. To address the class imbalance problem, a dual-loss strategy combining cross-entropy loss and Dice loss was employed, which enhances the segmentation performance of minority classes and improves overall robustness and generalization.

The remainder of this paper is organized as follows: Section 2 presents the dataset construction and preprocessing procedures; Section 3 describes the architecture and components of the proposed model; Section 4 outlines the experimental setup and performance evaluation; Section 5 discusses the main findings and their implications; and Section 6 concludes the study and suggests directions for future work.

## 2. Study Area and Data Sources

### 2.1. Study Area

**Figure 1:**
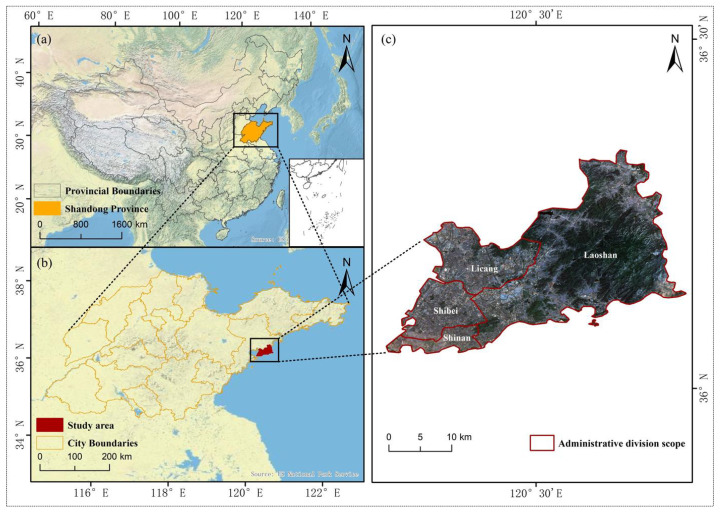
Schematic map of the main urban study area in Qingdao, Shandong Province, China. (**a**) The location of Shandong Province (orange) within China. (**b**) The position of the study area (dark red) within Shandong Province. (**c**) A satellite image showing the main urban districts of Qingdao, including Shinan, Shibei, Licang, and Laoshan. The administrative boundaries of each district are outlined in red.

Qingdao is situated in the southern part of the Shandong Peninsula, bordered by the Yellow Sea to the east. It is recognized as an important coastal center, coastal resort, tourist destination, and international port city in China, covering a total area of 11,293 km^2^. The study area of the paper was selected as the main urban area of Qingdao, which includes the Shinan District, the Shibei District, the Laoshan District, and the Licang District, as shown in Figure 1. The area is characterized by a high population density and complex urban functional layout, and at the same time has rich urban green space resources, such as urban parks, street green belts, wetlands and other types of green space. The topography of the region generally exhibits a trend of high elevations in the east and low elevations in the west, with complex and diverse terrain. The region experiences a temperate monsoon climate with an average annual temperature of around 12 °C. The four seasons are distinct, with summers being hot, humid, and rainy, and winters being cold and dry.

### 2.2. Data Preparation

Common urban green space types in Qingdao can be divided into three major categories: deciduous trees, evergreen trees, and grasslands. Among them, deciduous trees mainly include ginkgo, French sycamore, willow, cherry tree, maple, acacia, elm, poplar, ash, persimmon, etc. Evergreen trees include cedar, black pine, cypress, chaste tree, heather, magnolia, osmanthus, Nanyang fir, cypress, balsam fir, etc., and grassland includes natural grassland and artificial grassland. The specific remote sensing interpretation signs are shown in Table 1 [46].

### 2.3. Dataset Construction

**Figure 2:**
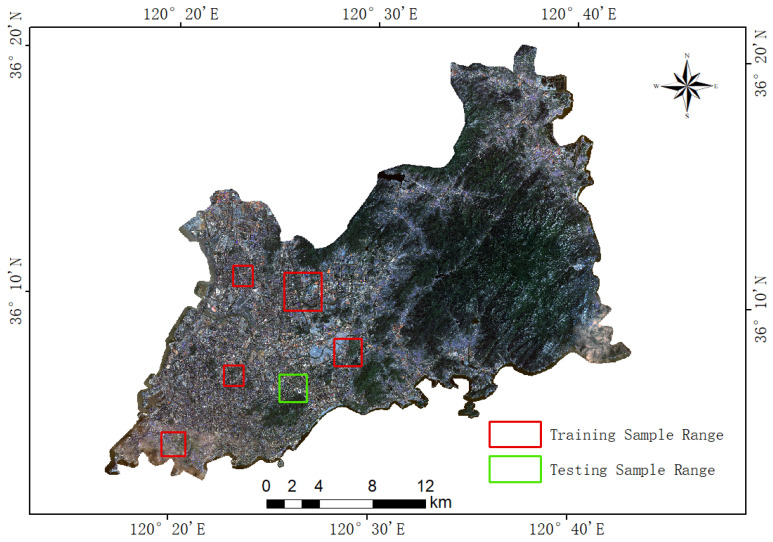
Distribution map of training and testing samples selection.

In this paper, domestic Gaofen-2 (GF-2) satellite multispectral imagery was employed as the primary research data, with data acquired from the China Resource Satellite Application Center. GF-2 is China’s first independently developed civilian optical remote sensing satellite with a spatial resolution better than 1 m. It is equipped with a Panchromatic and Multispectral Sensor (PMS), providing a spatial resolution of 1 m in the panchromatic band and a resolution of 4 meters in the multispectral bands. The imagery used in this paper features a spatial resolution of 1 m (after pansharpening). To ensure image quality and meet research requirements, a series of preprocessing operations, including radiometric calibration, atmospheric correction, orthorectification, and cloud removal, were performed on the raw images. Standard false-color composites generated from bands 4, 3, and 2 were utilized to produce 1-m resolution remote sensing images for the summers and winters of 2023. Urban green space classification requires substantial labeled training data related to green space targets; however, most existing publicly available datasets are designed for land cover mapping, and many urban green space datasets created from high-resolution imagery are not open-source, limiting their applicability for deep learning tasks. Therefore, in this paper, Google Earth imagery and winter GF-2 images were used primarily as reference data to support the annotation of summer GF-2 imagery, and six representative urban green space areas were selected based on field surveys. These areas covered typical types of urban green spaces, including centrally distributed urban parks (e.g., Signal Hill Park), sporadically distributed residential green spaces (e.g., residential areas in Licang District), and regularly distributed artificial green spaces (e.g., Qingdao International Golf Course). The selection of these regions aimed to reduce data redundancy, enhance analysis efficiency, and provide rich and diverse samples for subsequent model training and validation. The locations of the selected regions are presented in Figure 2.

**Figure 3:**
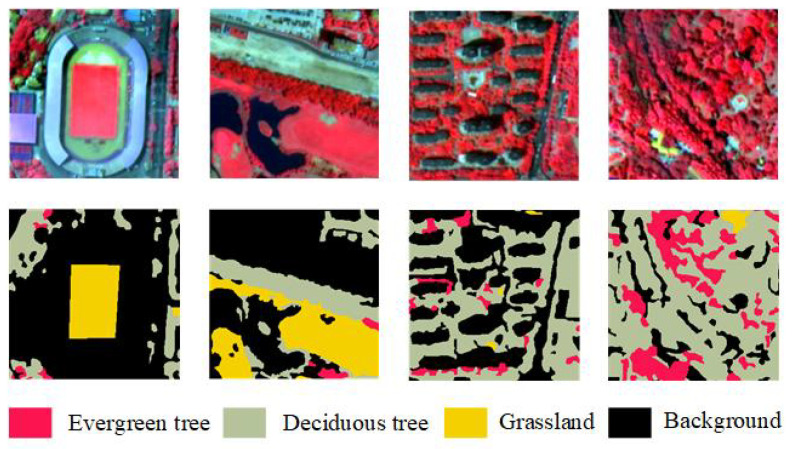
Sample GF-2 image patches and their corresponding ground truth segmentation annotations. The top row shows four representative false-color GF-2 remote sensing images from the constructed dataset. The bottom row displays the corresponding manually labeled ground truth maps, annotated with four vegetation classes: evergreen trees (red), deciduous trees (gray-green), grassland (yellow), and background (black).

The contours of green space types were delineated through manual visual interpretation and were corrected using field survey data. Raster images were subsequently generated as ground truth labels. The images and corresponding labels were cropped into 256 × 256 pixels using the sliding window method. To address problems such as overfitting caused by the limited number of samples, a series of data enhancement operations, such as flipping, rotation, and translation, were performed on the samples in the paper. The dataset was divided into training, validation, and test sets in a ratio of 6:2:2, ensuring that no overlap existed among the three sets. The final constructed sample set comprised 1768 images of 256 × 256 pixels. A sample example is presented in Figure 3.

## 3. Modeling Methodology

The DeepLab family is a series of deep learning models developed by Google [25,26], specialized in semantic segmentation tasks of images and widely used in several scenarios due to their excellent performance. The DeepLabv3+ model adopts an encoder–decoder structure, and the encoder part uses Xception [47] and ResNet50 as the backbone network to capture deep features in complex scenes by acquiring multi-scale global contextual information through a hollow space pyramid pooling (ASPP) module using dilated convolutions. The decoder structure is designed to compensate for the missing information caused by the subsampling process and to fuse the low-level features extracted from the encoder, thus improving the segmentation of object boundaries while maintaining high accuracy. With its efficiency and accuracy, this model is widely used in image segmentation tasks for natural scenes [48,49,50].

### 3.1. Contextual Transformer Module

**Figure 4:**
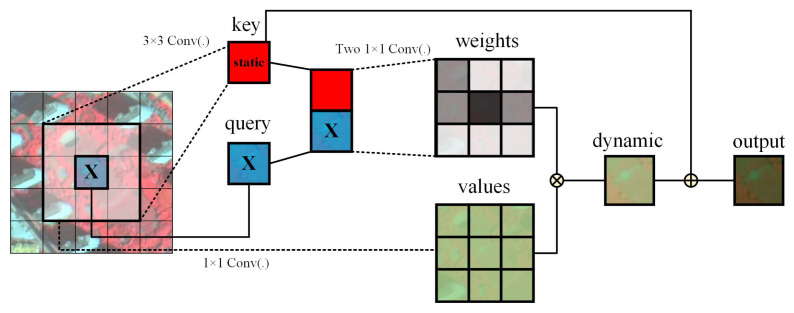
Schematic diagram of static and dynamic context information extraction and fusion in Contextual Transformer.

The DeepLabV3+ model relies primarily on traditional convolutional operations to extract features, but the local sensory field of traditional convolutional operations limits the ability of the model to capture long-distance dependencies, especially in complex or large-scale scenes. The self-attention mechanism can reduce dependence on external information and excels at capturing the internal correlation of features, which can effectively solve the long-distance dependency problem. However, the traditional attention mechanism, which learns independently through isolated query key pairs, fails to explore the rich contextual information in between, a feature that limits its feature learning capability. To address these limitations, this paper introduces the CoT module in the decoder part. This module can capture the input static contextual information as well as promote the learning of dynamic contextual information by context-coding adjacent key pairs. It integrates static and dynamic contextual information into the same architecture to fully utilize the contextual information of adjacent pixels, thus strengthening global information extraction and characterization, and finally capturing the overall morphology and distribution of urban green spaces. The structure of the CoT module is shown in Figure 4.

Given the input features $X\in R^{H\times W\times C}$, compute the Query, Key, and Value matrices:

(1) $$Q=X,\;K=X,\;V=XW_{v}$$

where $W_{v}$ denotes a 1×1 dynamic convolution, and *V* is the resulting value feature. Although *Q* and *K* are both derived from the input feature map *X*, they serve different purposes in the CoT module. Specifically, *K* is processed with a 3×3 convolution to extract local spatial context and produce static contextual features, while *Q* preserves the original feature information and is concatenated with the contextualized *K* to generate dynamic attention weights.

Secondly, the static interaction processing is realized by applying k×k group convolution to all neighboring key values in the k×k grid to obtain the static feature information $K_{1}$ of the input feature map *X*. Then, the static context information $K_{1}$ and query matrix *Q* are concatenated and processed by two consecutive convolution layers to obtain the attention weight matrix *A*:

(2) $$A=\left[K_{1},Q\right]W_{\theta }W_{\delta }$$

where $W_{\theta }$ is the convolution layer with activation (e.g., ReLU), and $W_{\delta }$ is without activation.

Next, the dynamic context feature $K_{2}$ is obtained by applying dot-product attention between *A* and *V*:

(3) $$K_{2}=A⊗V$$

Finally, the output of the CoT module is produced by summing the static and dynamic feature representations:

(4) $$Y=K_{1}+K_{2}$$

### 3.2. SENetv2 Attention Mechanism

**Figure 5:**
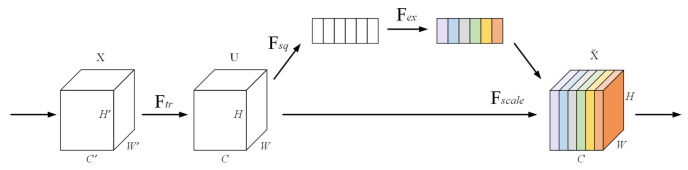
Structure of the SE module. The input tensor *X* undergoes sequential squeezing ($F_{\mathrm{sq}}$) and excitation ($F_{\mathrm{ex}}$) operations, and is subsequently scaled ($F_{\mathrm{scale}}$) to recalibrate the feature maps.

**Figure 6:**
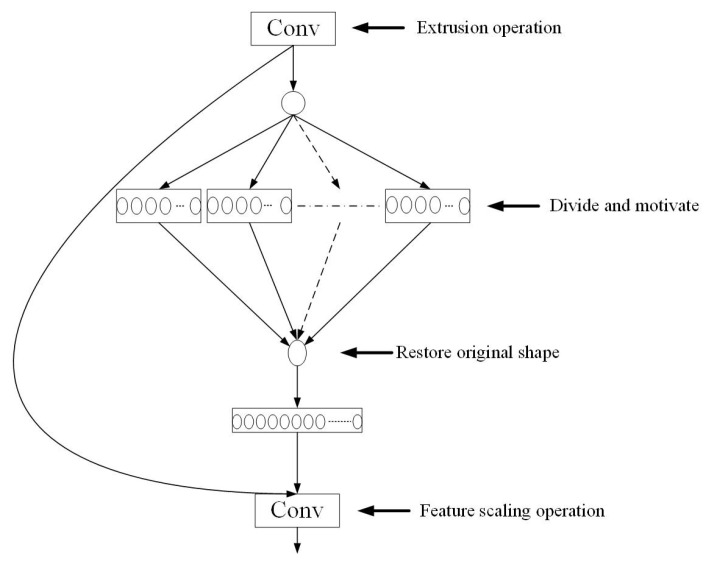
Structure of the Squeeze Aggregated Excitation module. The module starts with a global average pooling to extract channel-wise statistics, which is followed by a 1 × 1 convolution (“Extrusion operation”) to compress the features. The resulting descriptor is processed through multiple parallel fully connected branches (“Divide and motivate”) to generate aggregated excitation features. These features are concatenated and projected back to the original shape (“Restore original shape”). A final 1 × 1 convolution (“Feature scaling operation”) is applied to refine the recalibrated features.

The DeepLabV3+ model recovers the boundary and detailed structure of the target in the segmentation task by fusing deep and shallow features in the decoder part, which fully utilizes the semantic information of the deep features with detailed information of the shallow features. In this decoder inoculation, there is a lack of an explicit weighting mechanism between the feature channels, which makes it difficult to highlight the key channel features effectively. In particular, the limited processing power in the detail region often leads to problems such as blurred boundaries. To solve this problem, this paper introduces the SENetv2 attention mechanism into the decoder, which adds the Squeeze Aggregated Excitation (SaE) module [45], an aggregated dense layer on top of SENetv1 (the network structure of SENet is shown in Figure 5). The SaE module is based on the SE module [51], which aggregates a multi-branch fully connected (FC) layers for channel weighting. This enhances the model’s ability to focus on channels and improves the extraction of important features while suppressing irrelevant and redundant ones. In addition, the SaE module is also optimized in terms of computational efficiency, which is suitable for application to high spatial resolution feature maps. Its structure is shown in Figure 6.

Given the input feature $X\in R^{H\times W\times C}$, the global statistical information of each channel is first extracted by global average pooling (squeezing operation). Then, the weights of the channels are computed through a multi-branch fully connected layer (excitation operation), which generates the importance weights of each channel and multiplies these weights with the original features channel by channel to achieve the re-weighting of the feature map, retaining the key features and suppressing irrelevant or redundant information. The output is shown in Equation (5):

(5) $$\mathrm{SENetv}2=X+F\left(X\cdot \mathrm{Ext}\left(\sum \mathrm{Sq}(X)\right)\right)$$

where ∑Sq(X) is the compression operation after aggregation, Ext is the excitation operation, and F(·) denotes the standard set of operations in the SaE module, which includes normalization and dropout.

### 3.3. CTSA-DeepLabV3+ Model Construction

**Figure 7:**
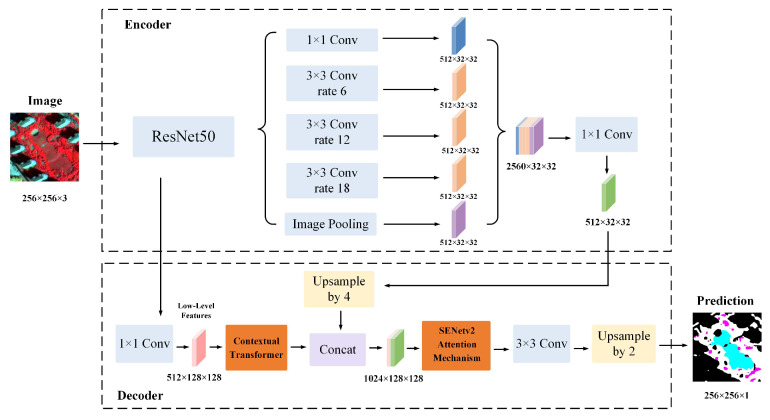
Flowchart of the improved DeepLabV3+ model.

The refined segmentation model CTSA-DeepLabV3+ is proposed based on the DeepLabv3+ network structure. In the encoder structure, ResNet50 is chosen as the backbone network, which is able to maintain a better feature extraction capability while ensuring a lower computational cost. In the decoder part, the CoT module and SENetv2 attention mechanism are introduced. Among them, the CoT module can better capture complex spatial patterns and global context information, which effectively improves the segmentation accuracy and boundary detail processing ability. The CoT module is applied to shallow features, which enables the model to better handle boundary details in urban green space images, especially in complex urban environments, and the module plays a crucial role. In addition, the SENetv2 attention mechanism is integrated into the decoder part of DeepLabV3+ for channel weighting processing after the fusion of deep and shallow features. By adaptively weighting the channel features, the improved model is able to focus on the key features more accurately, which can significantly enhance the ability of the model to detect small areas of green space as well as its ability to process detailed areas. Especially in complex urban environments, the SENetv2 attention mechanism effectively reduces the fragmentation of green space boundary delineation and further improves the accuracy of the model in classifying green spaces. In addition, through the adaptive recalibration mechanism, the SENetv2 attention mechanism improves the accuracy and robustness of the model, ultimately enhancing its overall performance in urban green space classification. In addition, in the decoder, the feature map is upsampled using a bilinear interpolation method to recover to the same spatial resolution as the input image. With better smoothness and lower computational overhead, this method can effectively retain the spatial detail information extracted in the coding stage and improve the spatial continuity of the final segmentation result. Finally, for the category imbalance problem in the urban green space classification task, both the cross-entropy loss function and the Dice loss function were used to improve the overall segmentation accuracy of the model. The network structure diagram of the improved model is shown in Figure 7.

### 3.4. Evaluation Metrics

In order to quantitatively evaluate the precision performance of the improved model in the classification task, the paper uses a variety of commonly used precision evaluation metrics, including mean intersection over union (MIoU), overall accuracy (OA), precision, recall, and F1-Score. The definitions of each evaluation metric are presented as follows:

(6) $$\mathrm{MIoU}=\frac{1}{K_{c}+1}\sum_{i=0}^{k}\frac{P_{ii}}{\sum_{j=0}^{k}P_{ij}+\sum_{j=0}^{k}P_{ji}-P_{ii}}$$

where $K_{c}$ represents the number of classes; $P_{ii}$ represents the number of correctly classified pixels; $P_{ij}$ represents the number of pixels of class *i* misclassified as class *j*; and $P_{ji}$ represents the number of pixels of class *j* misclassified as class *i*.

(7) $$\mathrm{OA}=\frac{TP+TN}{TP+TN+FP+FN}$$

(8) $$\mathrm{Precision}=\frac{TP}{TP+FP}$$

(9) $$\mathrm{Recall}=\frac{TP}{TP+FN}$$

(10) $$F1=2\times \frac{\mathrm{Precision}\times \mathrm{Recall}}{\mathrm{Precision}+\mathrm{Recall}}$$

where TP (True Positive) is the number of pixels correctly predicted as belonging to the target category, TN (True Negative) is the number of pixels correctly predicted as not belonging to the target category, FP (False Positive) is the number of pixels incorrectly predicted as belonging to the target category, and FN (False Negative) is the number of pixels incorrectly predicted as not belonging to the target category.

In the context of urban green space classification, each category (e.g., evergreen trees, deciduous trees, grassland, and background) is treated as an independent binary classification task. TP, TN, FP, and FN are calculated separately for each category to comprehensively evaluate the model’s performance.

## 4. Results Analysis

### 4.1. Training Setup of the Model

The experiments were conducted using the PyTorch (PyTorch 1.10.0+cu113) deep learning framework. The computer hardware consisted of an Intel(R) Core(TM) i7-12700H processor (Intel(R): Santa Clara, CA, USA) and an NVIDIA GeForce RTX 3060 graphics card (NVIDIA: Santa Clara, CA, USA). The environment was based on Python 3.8 and CUDA 11.0, meeting the training requirements for semantic segmentation models. The Adam optimizer was selected, with the batch size set to 16. The maximum number of training epochs was set to 10,000, the initial learning rate was set to $1\times 10^{-3}$, and the learning rate adjustment strategy adopted a polynomial decay (poly policy) with a decay exponent of 0.9 and a minimum learning rate (min_lr) of $1\times 10^{-4}$. The learning rate was dynamically adjusted based on the number of iterations to ensure convergence and optimal final performance during training.

### 4.2. Model Performance Evaluation

To verify the effectiveness of the CTSA-DeepLabV3+ model for urban green space classification, comparative experiments were conducted between it and several mainstream semantic segmentation models in the paper. Five models—FCN, UNet, PSPNet, DeepLabv3+, and UperNet-Swin Transformer—were selected as baselines to comprehensively evaluate classification accuracy, result quality, computational efficiency, and automation level. To facilitate visual comparison, local features from five typical areas were extracted for analysis (Table 2). The results demonstrated that FCN, UNet, PSPNet, UperNet-Swin Transformer, and the original DeepLabv3+ models exhibited notable limitations in green space classification. Although these models distinguished urban green spaces from non-green spaces, omissions and misclassifications were frequently observed in green space type classification, such as evergreen trees being misclassified as deciduous trees, inaccurate edge segmentation, and jagged boundaries, particularly in detail-rich and complex scenes. Specifically, FCN struggled to distinguish complex green space types due to resolution degradation and feature loss from multiple subsampling. UNet, despite strong local feature extraction capability, failed to capture sufficient global context, leading to blurred boundaries in textured scenes. PSPNet, while enhancing contextual information via multi-scale feature fusion, produced overly smooth edges and lacked detailed classification accuracy. UperNet-Swin Transformer suffered from limited local feature representation in complex scenes, resulting in significant discrepancies from ground truth labels. The original DeepLabv3+ model showed weak boundary detail extraction, with boundary blurring and missing features. In contrast, the CTSA-DeepLabV3+ model enhanced contextual expression through the CoT module, effectively captured fine details, and reduced feature loss and misclassification. Moreover, the integration of the SENetv2 attention mechanism optimized boundary processing, mitigated blurring and jaggedness, and clarified boundaries between green spaces and other categories, resulting in significant overall improvement.

Table 3 shows the accuracy comparison between the paper’s method and the other five deep learning models for urban green space classification. It can be seen that the CTSA-DeepLabV3+ model achieves significant improvement in all evaluation indexes, which is significantly higher than the other models. Specifically, the MIoU, OA, precision, recall, and F1-score of the CTSA-DeepLabV3+ model reach 89.22%, 96.21%, 92.56%, 90.12%, and 91.23%, respectively, which is the best performance among all the methods. Compared with FCN, UNet, PSPNet, UperNet-Swin Transformer, and the original DeepLabV3+ model, the overall accuracy of this model improved by 1.32%, 1.18%, 1.68%, 1.85%, and 1.08%, respectively. The CTSA-DeepLabV3+ model not only achieves a significant increase in the overall classification accuracy of the MIoU, precision, recall, and F1-score and other accuracy evaluation metrics are also significantly better than the comparison model, which indicates that it has stronger robustness and accuracy in complex scenarios and multi-category classification tasks.

To evaluate computational efficiency and performance in urban green space classification, the computational complexity (FLOPs), parameter count (Parameters), and average inference time (Inference Time) of different models were compared in this paper, as shown in Table 4. On 256 × 256 pixel test images, the CTSA-DeepLabV3+ model has the highest computational complexity, with 123.02 G FLOPs, 118.94 M parameters, and 153.31 ms inference time. In comparison, DeepLabV3+ (44.06 G, 41.22 M, 61.20 ms) and PSPNet (44.66 G, 46.61 M, 61.13 ms) offer better computational efficiency, while FCN (72.31 G, 3.73 M, 81.60 ms) has higher computational demand but fewer parameters. UperNet-Swin Transformer (59.65 G, 58.94 M, 74.39 ms) has higher computational complexity due to its Transformer structure, but it enhances long-range dependency modeling. UNet has the lowest computational complexity, with only 1.94 G FLOPs, 1.87 M parameters, and the fastest inference time (6.02 ms), but its smaller parameter size and shallow network structure may limit its classification accuracy on high spatial resolution remote sensing images.

Despite the moderate increase in computational overhead, the CTSA-DeepLabV3+ model demonstrated superior classification accuracy, fine-grained feature extraction, and improved boundary delineation, attributed to the CoT module’s global context modeling and the SENetv2 module’s feature enhancement. These improvements significantly enhanced the generalization ability and robustness of the model, making it well-suited for tasks requiring fine classification, such as high-precision remote sensing interpretation and urban ecological monitoring.

### 4.3. Ablation Experiment

The improvement of the DeepLabV3+ model in the paper focused on two aspects: the introduction of a CoT module and the SENetv2 attention mechanism. The improvement effect of the CoT module and SENetv2 attention mechanism on urban green space remote sensing classification is verified by ablation experiments on the CTSA-DeepLabV3+ model, and the experimental results are shown in Table 5. The experimental results show that all evaluation indexes of the original DeepLabV3+ model are low. By introducing the CoT module or the SENetv2 attention mechanism, respectively, the model is significantly improved in terms of classification accuracy and detail processing ability. When the CoT module was introduced, the OA, MioU, precision, recall, and F1-score of the model were improved by 0.49%, 1.22%, 0.92%, 0.93%, and 0.97%, respectively. When the SENetv2 attention mechanism is introduced, the accuracy of the model is further improved by 0.51% and 1.5% for OA and MIoU, and 1.43%, 1.17%, and 1.27% for precision, recall, and F1-score, respectively. When the CoT module and SENetv2 attention mechanisms were used jointly, all the evaluation metrics of the model reached a high level, with OA and MIoU improving by 1.08% and 3.02%, and precision, recall, and F1-score improving by 3.29%, 2.38%, and 2.84%, respectively. This indicates that the synergy between the CoT module and the SENetv2 attention mechanism further enhances the model performance, which effectively improves the classification of urban green spaces in complex scenes.

Table 6 shows the urban green space classification results of typical regional ablation experiments. From the visualization effects, significant contributions were observed from the CoT module and SENetv2 attention mechanism in enhancing global feature capture and detail extraction capabilities of the model. The CoT module captures the dynamic and local detail information of the neighboring keys through the context encoding mechanism, optimizes the learning of the attention matrix, and strengthens the visual recognition ability of the model, especially in processing complex boundary regions and texture details, thereby enabling accurate capture of boundary features and improving the overall classification accuracy. The SENetv2 attention mechanism realizes adaptive weighting of channel features through the multi-branch fully connected layer, effectively highlighting key channel information and weakening redundant interference. When it acts on deep and shallow layer feature fusion, it significantly optimizes the feature synergy ability, makes the boundary segmentation smoother, the detail classification more accurate, and reduces the phenomenon of omission and misclassification. The classification results are further improved when the CoT module and SENetv2 attention mechanism are used jointly, and the two significantly enhance the adaptability and robustness of the model in complex greenfield scenes through the synergy of global context modeling and channel feature aggregation. The improved model performs well in classifying highly textured regions and complex boundaries with smooth boundaries. This suggests that the combination of the CoT and SENetv2 attention mechanism effectively improves the segmentation details and overall performance of the urban green space classification task.

### 4.4. Urban Green Space Classification Results

**Figure 8:**
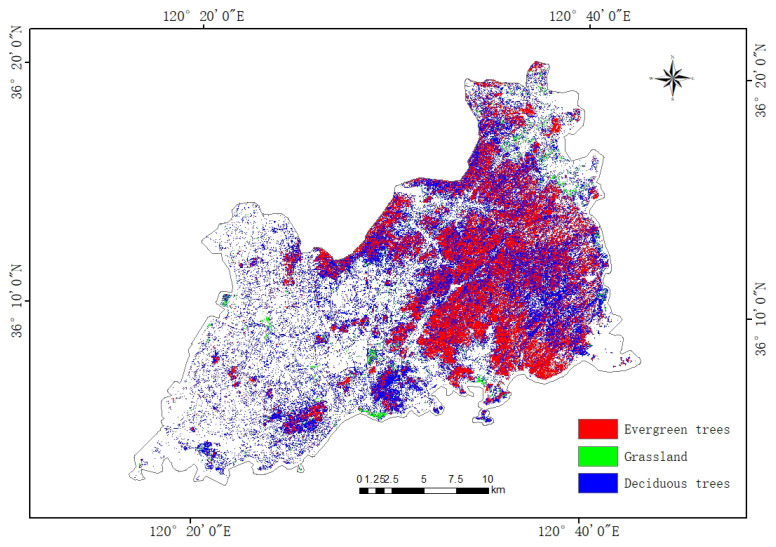
Urban green space classification map of the main urban area of Qingdao.

Through the above experiments, it is proved that the CTSA-DeepLabV3+ model proposed in this paper has a good classification effect on urban green space classification of high spatial resolution remote sensing images, and at the same time, it shows good model generalization ability and migration learning potential. On this basis, the paper’s urban vegetation classification system is adopted to classify the urban green space in the main urban area of Qingdao city. Its spatial distribution is shown in Figure 8. The area of different urban green space categories was calculated using the ArcGIS platform, and the statistical results are shown in Table 7.

The classification results show that the urban green space in the main urban area of Qingdao presents obvious spatial heterogeneity characteristics. Deciduous trees are mainly distributed in the central area of the city, covering the largest area of about 154.41 km^2^, indicating that seasonal vegetation dominates the urban green space system. Evergreen trees cover an area of 120.20 km^2^, mainly concentrated in the southern and southeastern regions, which is important for maintaining the city’s perennial greening and ecological stability. In contrast, the area of grassland is only 7.50 km^2^, which accounts for a relatively small proportion of the total green space in the city and is mainly distributed in the form of scattered patches.

The above results show that deciduous trees dominate the urban greening structure in the main urban area of Qingdao and are widely distributed. The spatial clustering characteristics of different green space types not only reflect the urban greening planning preferences but also are influenced by topographic conditions, land use history, and microclimate environment. The model proposed in this paper can accurately depict the spatial distribution of various types of green space in complex urban environments, better reflecting the distribution characteristics of urban green space in Qingdao’s main urban area, which is a blend of “mountain-sea-city”, and provides a reliable data basis for subsequent urban ecological assessment and green space system planning.

## 5. Discussion

### 5.1. Generalization Capability of the Improved Model in Handling Complex Images

To systematically assess the classification capability of CTSA-DeepLabV3+ across varying levels of scene complexity, this paper selected representative regions with distinct characteristics from the GF-2 dataset. These regions covered simple scenarios (such as large, homogeneous green areas), moderately complex scenarios (such as areas with interspersed vegetation and buildings), and highly complex scenarios (such as fragmented green spaces intersected by roads and areas significantly affected by shadow occlusion). Comparative analysis across these scene types confirmed the robustness and adaptability of CTSA-DeepLabV3+ in handling diverse urban environments, as detailed in Table 8.

In simple scenarios, all models were able to accurately identify large, continuous green spaces. However, CTSA-DeepLabV3+ achieved superior performance in handling boundary details, producing clearer and more precise segmentation along edges. Due to the homogeneous texture and singular targets in such scenes, the model effectively leveraged the global context modeling capability of the CoT module, combined with the optimized spectral feature selection enabled by the SENetv2 attention mechanism, to achieve high-precision classification.

In moderately complex scenes, classification results showed frequent confusion between evergreen and deciduous trees, indicating that the model’s ability to distinguish vegetation with similar spectral characteristics still requires further improvement. In highly complex scenarios—such as those characterized by fragmented green patches, mixed distributions of vegetation, buildings, and roads, and severe shadow occlusion—the classification task proved most challenging. Although the model exhibited more frequent misclassifications in such environments, it significantly reduced issues related to blurred boundaries and segmentation fragmentation compared to baseline models. Further analysis revealed that the CoT module effectively captured the spatial distribution patterns of fragmented green spaces through dynamic contextual encoding, while the SENetv2 attention mechanism successfully suppressed interference from non-vegetation features such as building shadows.

These experimental findings demonstrate that CTSA-DeepLabV3+ approaches near-optimal performance in simple scenarios. However, its accuracy moderately declines in more complex environments as a result of increased target diversity and background interference. This degradation is primarily caused by spectral confusion induced by mixed pixels in high-resolution imagery, as well as greater morphological variability in small-scale green spaces, both of which increase the difficulty of feature representation and learning. Despite these challenges, the model exhibits enhanced robustness through several mechanisms: (i) multi-scale context fusion in the CoT module mitigates detail loss at complex boundaries; (ii) channel reweighting in SENetv2 improves inter-class feature discrimination; and (iii) the combined use of cross-entropy and Dice loss functions helps address class imbalance issues during training.

### 5.2. Generalization Capability of the Improved Model Across Different Geographic Regions

**Figure 9:**
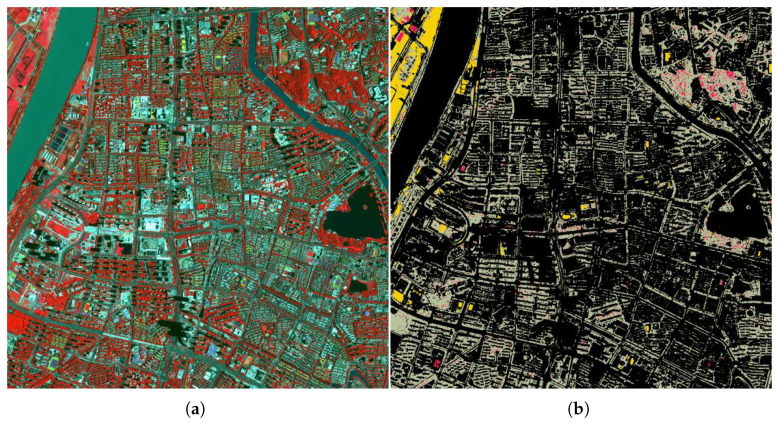
GF-2 imagery and corresponding urban green space classification results for the selected area in Nanjing. (**a**) Original GF-2 satellite image. (**b**) Classification results generated by the proposed model.

To further evaluate the generalization capability of the model under varying geographic conditions, this study selected several urban areas in Nanjing—characterized by distinct spatial structures of urban green spaces and different geographic locations—as test regions. The model was directly transferred to GF-2 imagery of Nanjing without retraining, and its performance was systematically assessed in terms of classification accuracy and boundary delineation quality. This experiment aimed to validate the model’s generalizability across urban environments with diverse regional characteristics, see Figure 9.

The test results indicate that the improved model exhibits good generalization capability in classifying green space types in the urban areas of Nanjing. Overall, the spatial distribution patterns of the three green space categories are clearly delineated. In particular, the model accurately identified green boundaries and effectively distinguished between vegetation types in areas such as urban parks, residential green spaces, and institutional green areas. Evergreen trees were mainly concentrated in park core zones and along certain roadside green belts. The model achieved stable segmentation performance for this class, with strong edge continuity and well-defined boundary contours. Deciduous trees, which had a wider spatial distribution, were effectively segmented into streets, residential areas, and open green zones. The model successfully differentiated them from adjacent grasslands and evergreen vegetation. Grasslands were primarily located in open urban spaces and along riverfront landscapes. The segmentation results were largely consistent with the actual spatial layout, and the classification boundaries showed smooth transitions, reflecting a high degree of spatial coherence.

However, some confusion between deciduous and evergreen trees persisted in shadow-covered areas of high-density urban blocks, especially in zones with densely interlaced green belts, likely due to spectral mixing effects that caused boundary ambiguity. In addition, misclassification between grass and deciduous trees occasionally occurred in regions with exposed soil or low-stature herbaceous vegetation, indicating that the model still encounters challenges in fine-grained classification when applied to cross-regional samples.

These findings confirm that the proposed model demonstrates strong transferability and high classification accuracy in the Nanjing region. It effectively adapts to variations in urban green space structures and spectral characteristics across different geographic contexts, underscoring its robust regional generalization performance.

## 6. Conclusions

A high spatial resolution urban green space sample dataset was constructed based on GF-2 remote sensing images, and the CTSA-DeepLabV3+ model was proposed by improving the traditional DeepLabV3+ structure to achieve more efficient, accurate, and intelligent urban green space classification. In the improved model, the CoT module and SENetv2 attention mechanism were integrated into the decoder, alleviating the mismatch between the encoder and decoder structures, capturing global contextual information, optimizing channel feature representation, and enhancing classification performance.

Experimental results show that the CTSA-DeepLabV3+ model achieved an overall accuracy (OA) of 96.21%, a mean intersection over union (MIoU) of 89.22%, and precision, recall, and F1 scores of 92.56%, 90.12%, and 91.23%, respectively. Compared with five representative baseline models (FCN, U-Net, PSPNet, DeepLabV3+, and UperNet-Swin Transformer), the proposed model consistently outperformed them in all accuracy metrics. Moreover, the model demonstrated strong capability in classifying complex and heterogeneous urban green spaces, accurately distinguishing between multiple vegetation types such as evergreen trees, deciduous trees, and grasslands, thus substantially improving multi-class discrimination. Although the introduction of attention modules led to a slight increase in computational complexity and inference time, this trade-off resulted in better classification accuracy, stronger fine-grained feature extraction, and improved boundary delineation, while still maintaining relatively high inference efficiency. These advantages make the model well-suited for high-resolution remote sensing image analysis. Furthermore, validation in a geographically distinct test area—urban Nanjing—confirmed that the model retained high classification accuracy and boundary recovery performance, further demonstrating its strong regional generalization ability.

However, the current model remains sensitive to the quality of remote sensing imagery, and low-quality inputs may adversely affect classification accuracy. Future research will consider integrating multi-source remote sensing data to enhance the model’s robustness under varying data conditions. In addition, incorporating richer auxiliary feature information into the deep learning framework is planned to further advance urban green space classification toward greater precision and intelligence.

## Figures and Tables

**Table 1:** Remote sensing interpretation indicators corresponding to common vegetation types in Qingdao.

Category	Species	Interpretation Features	Standard False Color
Deciduous Trees	Ginkgo, Willow, Maple, etc.	Appear with a red hue in remote sensing images, with rough texture.	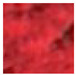
Evergreen Trees	Cedar, Black Pine, Privet, etc.	Appear with a dark red hue in remote sensing images, with clear and orderly texture, and a relatively regular canopy shape.	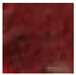
Grassland	Artificial Grass, Natural Grass	Appear with a light red hue in remote sensing images, with even and fine texture.	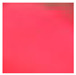

**Table 2:** Visualized comparative experimental results for typical regions.

	Image	Label	FCN	UNet	PSPNet	UperNet-SwinTransformer	DeepLabV3+	CTSA-DeepLabV3+
1	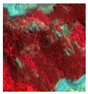	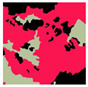	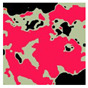	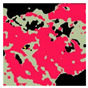	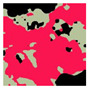	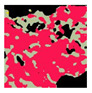	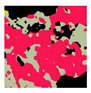	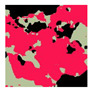
2	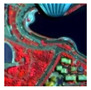	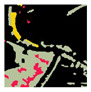	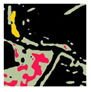	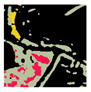	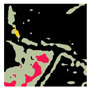	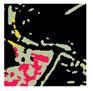	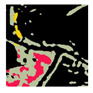	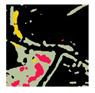
3	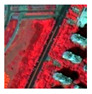	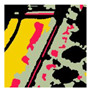	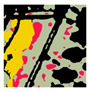	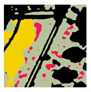	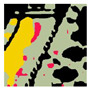	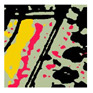	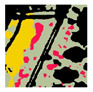	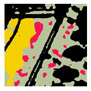
4	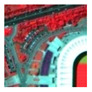	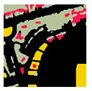	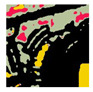	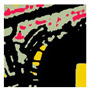	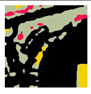	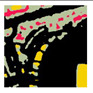	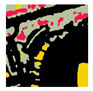	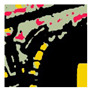
5	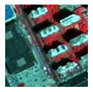	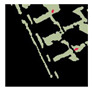	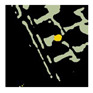	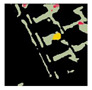	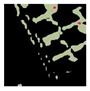	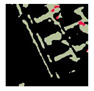	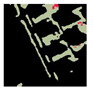	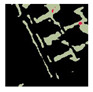

**Table 3:** Comparison of model accuracy (%).

Method	MIoU	OA	Precision	Recall	F1-Score
FCN	86.03	94.89	88.6	87.98	88.26
UNet	86.32	95.03	88.73	88.44	88.59
PSPNet	85.57	94.53	90.46	86.19	87.85
UperNet-Swin Transformer	84.49	94.36	88.54	85.41	86.51
DeepLabV3+	86.2	95.13	89.27	87.74	88.39
CTSA-DeepLabV3+	89.22	96.21	92.56	90.12	91.23

**Table 4:** Evaluation of model performance in terms of FLOPs, parameters, and inference time.

Models	FLOPs (G)	Parameters (M)	Average Inference Time (ms)	Input Size
FCN	72.31	3.73	81.60	256 × 256
UNet	1.94	1.87	6.02	256 × 256
PSPNet	44.66	46.61	61.13	256 × 256
UperNet-Swin Transformer	59.65	58.94	74.39	256 × 256
DeepLabV3+	44.06	41.22	61.20	256 × 256
CTSA-DeepLabV3+	123.02	118.94	153.31	256 × 256

**Table 5:** Accuracy results of the ablation study.

Method		Metrics (%)									
CoT	SENetv2	MIoU		OA		Precision		Recall		F1-Score	
		MIoU	Gain	OA	Gain	Precision	Gain	Recall	Gain	F1-Score	Gain
×	×	86.2	—	95.13	—	89.27	—	87.74	—	88.39	—
✓	×	87.44	+1.22	95.62	+0.49	90.19	+0.92	88.67	+0.93	89.36	+0.97
×	✓	87.7	+1.5	95.64	+0.51	90.7	+1.43	88.91	+1.17	89.66	+1.27
✓	✓	89.22	+3.02	96.21	+1.08	92.56	+3.29	90.12	+2.38	91.23	+2.84

**Table 6:** Visualized ablation study results for typical regions.

Method		Image		
CoT	SENetv2	Region 1	Region 2	Region 3
True Image		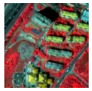	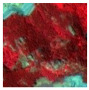	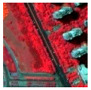
Label		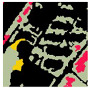	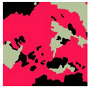	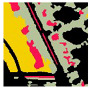
×	×	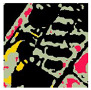	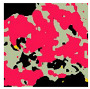	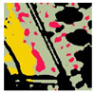
✓	×	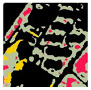	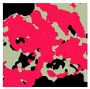	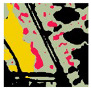
×	✓	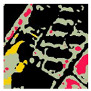	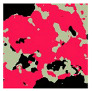	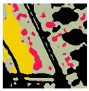
✓	✓	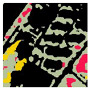	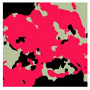	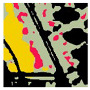

**Table 7:** Area statistics of different urban green space categories in Qingdao’s main urban area.

Category	Area (km^2^)
Deciduous tree	154.41
Evergreen tree	120.20
Grassland	7.50

**Table 8:** Visualization of CTSA-DeepLabV3+ classification results across urban green space scenes of varying complexity.

	Image	Label	FCN	UNet	PSPNet	UperNet-SwinTransformer	DeepLabV3+	CTSA-DeepLabV3+
1	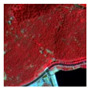	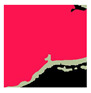	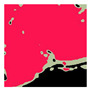	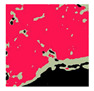	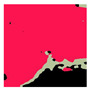	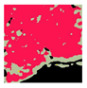	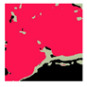	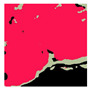
2	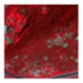	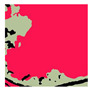	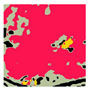	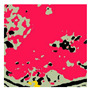	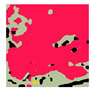	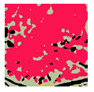	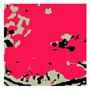	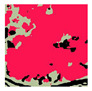
3	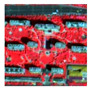	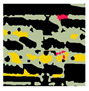	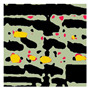	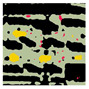	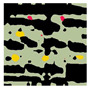	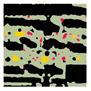	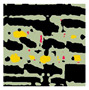	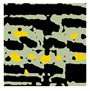
4	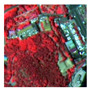	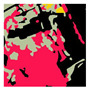	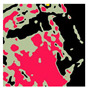	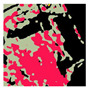	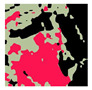	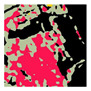	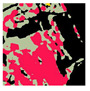	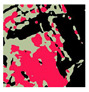
5	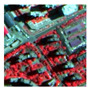	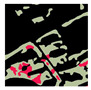	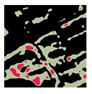	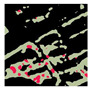	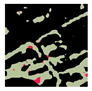	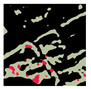	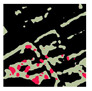	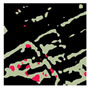
6	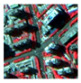	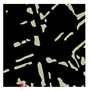	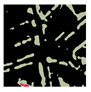	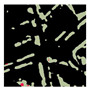	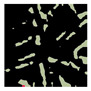	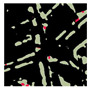	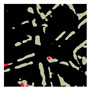	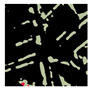

## Data Availability

The data presented in this study are available on request from the corresponding author.
